# Age-dependent atrial arrhythmic phenotype secondary to mitochondrial dysfunction in *Pgc-1β* deficient murine hearts

**DOI:** 10.1016/j.mad.2017.09.002

**Published:** 2017-10

**Authors:** Haseeb Valli, Shiraz Ahmad, Karan R. Chadda, Ali B.A.K. Al-Hadithi, Andrew A. Grace, Kamalan Jeevaratnam, Christopher L.-H. Huang

**Affiliations:** a Physiological Laboratory, University of Cambridge, Downing Street, Cambridge, CB2 3EG, United Kingdom; b Department of Biochemistry, University of Cambridge, Tennis Court Road, Cambridge, CB2 1QW, United Kingdom; c Faculty of Health and Medical Sciences, University of Surrey, GU2 7AL, Guildford, Surrey, United Kingdom; d PU-RCSI School of Medicine, Perdana University, 43400, Serdang, Selangor Darul Ehsan, Malaysia

**Keywords:** Peroxisome proliferator activated receptor-γ coactivator-1 (PGC-1), Atrial, Action potential, Wavelength, Cardiac conduction, Cardiac arrhythmias, Atrial fibrillation

## Abstract

**Introduction:**

Ageing and several age-related chronic conditions including obesity, insulin resistance and hypertension are associated with mitochondrial dysfunction and represent independent risk factors for atrial fibrillation (AF).

**Materials and methods:**

Atrial arrhythmogenesis was investigated in Langendorff-perfused young (3–4 month) and aged (>12 month), wild type (WT) and peroxisome proliferator activated receptor-γ coactivator-1*β* deficient (*Pgc-1β*^−/−^) murine hearts modeling age-dependent chronic mitochondrial dysfunction during regular pacing and programmed electrical stimulation (PES).

**Results and discussion:**

The *Pgc-1β*^−/−^ genotype was associated with a pro-arrhythmic phenotype progressing with age. Young and aged *Pgc-1β*^−/−^ hearts showed compromised maximum action potential (AP) depolarization rates, (d*V*/d*t*)_max_, prolonged AP latencies reflecting slowed action potential (AP) conduction, similar effective refractory periods and baseline action potential durations (APD_90_) but shortened APD_90_ in APs in response to extrasystolic stimuli at short stimulation intervals. Electrical properties of APs triggering arrhythmia were similar in WT and *Pgc-1β^−/−^* hearts. *Pgc-1β*^−/−^ hearts showed accelerated age-dependent fibrotic change relative to WT, with young *Pgc-1β^−/−^* hearts displaying similar fibrotic change as aged WT, and aged *Pgc-1β^−/−^* hearts the greatest fibrotic change. Mitochondrial deficits thus result in an arrhythmic substrate, through slowed AP conduction and altered repolarisation characteristics, arising from alterations in electrophysiological properties and accelerated structural change.

## Highlights

• Ageing and several chronic conditions are associated with mitochondrial dysfunction.
• We investigated atrial arrhythmia in energetically deficient Pgc-1β-/- murine hearts.
• Pgc-1β-/- hearts showed increased age-dependent arrhythmic and fibrotic changes.
• These were attributed to slowed action potential depolarization and conduction.
• The latter offer mechanisms for the atrial arrhythmia observed in these conditions.

## Graphical abstract

**Figure:**
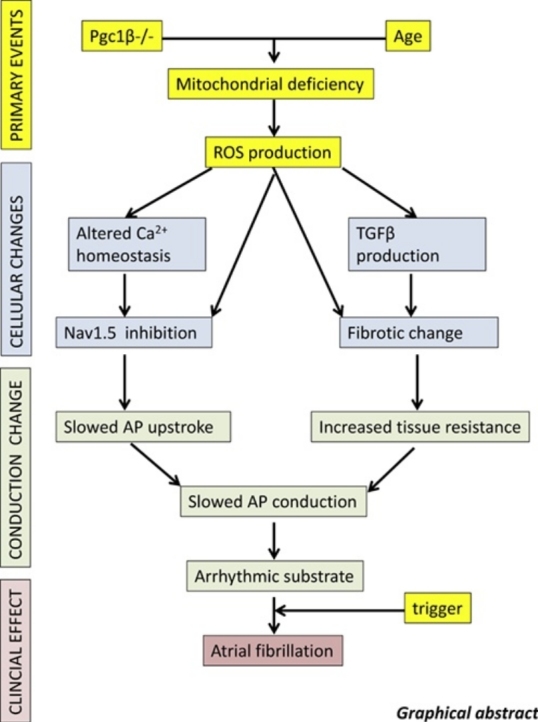

## 1 Introduction

Atrial fibrillation (AF) affects 1–3% of the population in the developed world (DeWilde et al., 2006, Friberg and Bergfeldt, 2013, Majeed et al., 2001) and is associated with significant morbidity and mortality, including five-fold increases in risks of stroke (Wolf et al., 1991) and significant increases in risks of all-cause mortality (Benjamin et al., 1998, Chamberlain et al., 2015, Friberg et al., 2007). Recent studies forecast a substantial increase in the incidence and prevalence of AF in the coming decades, with potentially 9 million cases in the United States (Miyasaka et al., 2006) and 18 million in Europe by 2060 (Krijthe et al., 2013), underpinning its recognition as a global epidemic. Its prevalence appears to a great extent to be a function of age, affecting 0.1% of individuals under the age of 55, reaching 20% of those above the age of 80 (Go et al., 2001, Zoni-Berisso et al., 2014). The aetiological significance of this age-related incidence or indeed the mechanisms underlying the initiation and perpetuation of AF remain incompletely explained. It is however clear that AF is a dynamic process, which at its inception is characterised by fleeting episodes of the abnormal rhythm triggered by focal ectopic activity in the pulmonary vein sleeves (Haïssaguerre et al., 1998). With time, these episodes become more protracted and eventually permanent through progressive electrical and structural remodeling ultimately producing a tissue substrate itself conducive to arrhythmia maintenance. Treatment in these latter stages is far less efficacious (Cappato et al., 2010), highlighting the need to target therapies to the upstream processes.

There is now growing appreciation for roles of metabolic, and in particular mitochondrial, dysfunction in the pathogenesis of AF. Mitochondrial dysfunction is a recognised feature of ageing (Sun et al., 2016) as well as a number of the constituents of the metabolic syndrome including obesity (Bournat and Brown, 2010), insulin resistance (Patti and Corvera, 2010) and hypertension (Dikalov and Ungvari, 2013), all recognised risk factors for AF (Menezes et al., 2013). Abnormal mitochondrial structure and function have been reported in animal models of AF (Ausma et al., 1997, Morillo et al., 1995). Moreover, analysis of cardiomyocytes from human patients with AF demonstrate increased DNA damage (Lin et al., 2003, Tsuboi et al., 2001), structural abnormalities (Bukowska et al., 2008) and evidence of impaired function (Ad et al., 2005, Lin et al., 2003). Whether the observed mitochondrial abnormalities are a cause or consequence of AF, and the mechanisms through which these changes occur remain unclear.

Disruptions in normal mitochondrial activity are known to be pro-arrhythmic, through reduced provision of ATP and/or aberrant production of reactive oxygen species (ROS), and therefore are a potential upstream mediator of arrhythmogenesis (Faivre and Findlay, 1990, Fosset et al., 1988, Manning et al., 1984). Much of this work has however been in the context of acute, profound mitochondrial impairment during ischaemia-reperfusion and focussed on mechanisms of ventricular arrhythmias. Interestingly, Chen & colleagues recently demonstrated increased ectopic activity, burst firing and shortening of the action potential duration (APD) in pulmonary veins and left atria of rabbit hearts subjected to ischaemia-reperfusion (Lin et al., 2012). Moreover, sustained arrhythmias are generally thought to occur through maladaptive changes in the electrophysiological properties of a tissue, promoting the formation of re-entrant circuits. Such would arise through slowed conduction of the depolarising wavefront and/or shortening of the effective refractory period (ERP). Indeed, time-dependent alterations in both atrial conduction (Gaspo et al., 1997a, Zheng et al., 2016) and repolarisation (Bosch et al., 1999, Daoud et al., 1996, Gaspo et al., 1997b) properties have been reported in animal models and human AF. Reductions in the inward sodium currents (*I*_Na_), a major determinant of conduction velocity (King et al., 2013a), have been reported secondary to excess mitochondrial ROS production (Liu et al., 2010). Gap junction activity is also known to be similarly sensitive to mitochondrial function (Li et al., 2016, Sovari et al., 2013) and may also contribute to conduction slowing. Mitochondrial impairment and cardiac oxidative stress in general are also recognised to reduce action potential durations and ERPs (Chen et al., 2007, Kurokawa et al., 2011, Lesnefsky et al., 1991), both of which favour re-entry and arrhythmia.

The electrophysiological sequelae of chronic mitochondrial dysfunction have however been challenging to study, confounded by the early and often terminal development of contractile dysfunction. However, such evaluation now appears feasible in murine models lacking members of the peroxisome proliferator activated receptor-γ coactivator-1 (PGC-1) family of transcriptional coactivators. The PGC-1 family include PGC-1α and PGC-1β, which though found with a reasonable degree of ubiquity, are preferentially expressed in tissues with high oxidative capacity such as the heart, brain and skeletal muscle (Riehle and Abel, 2012) and act as key regulators of mitochondrial mass and function (Finck and Kelly, 2006, Lin et al., 2005). In cardiac cells, the PGC-1 coactivators interact with nuclear respiratory factor-1, estrogen related receptor-α and peroxisome proliferator-activated receptor-α, leading to increased mitochondrial biogenesis (Huss et al., 2004, Vega et al., 2000). They also act to upregulate expression of nuclear and/or mitochondrial encoded mitochondrial proteins involved in the tricarboxylic acid cycle, fatty acid β–oxidation and components of the oxidative phosphorylation complex (Arany et al., 2005). PGC-1 protein expression itself is increased by upstream signals such as those arising from cold exposure and aerobic exercise, thereby serving as a link between cellular energy stores and external stimuli ultimately coordinating mitochondrial activity with cellular energy demands (Sonoda et al., 2007). Interestingly, their expression levels are found to be reduced in obesity, insulin resistance, type II diabetes mellitus and ageing, correlating with the mitochondrial dysfunction that is seen in these conditions and implicating it in the their pathogenesis (Dillon et al., 2012, Leone and Kelly, 2011, Mootha et al., 2003).

Murine hearts lacking either Pgc-1α or Pgc-1β do not develop cardiac failure under conditions of baseline activity. Homozygous deficiency of *Pgc-1α* is associated with a mild baseline cardiac phenotype with no overt contractile dysfunction, but cardiac failure does develop following transverse aortic banding (Arany et al., 2005). Similarly, genetic ablation of *Pgc-1β* does not appear to be detrimental to cardiac function at baseline, but adrenergic challenge elicits blunted rate responses potentially reflecting underlying electrophysiological abnormalities (Lelliott et al., 2006). Indeed, Langendorff-perfused *Pgc-1β^−/−^* murine hearts displayed features consistent with increased vulnerability to ventricular arrhythmia, with greater episodes of action potential alternans, a recognised harbinger for arrhythmia, and more frequent episodes of ventricular tachycardia during programmed electrical stimulation (Gurung et al., 2011). Isolated cardiomyocytes in the latter study showed altered patterns of ion channel expression, spontaneous diastolic Ca^2+^ transients, and pro-arrhythmic after-depolarisation events. The electrophysiological alterations and any associated change in arrhythmic propensity in the atria of these hearts have hitherto not been investigated.

The present study investigates the atrial electrophysiological properties secondary to chronic mitochondrial insufficiency in murine hearts lacking *Pgc-1β.* The phenotypic effects of such a mitochondrial deficit are likely to be cumulative, evolving with advancing age. Young and aged WT and genetically modified hearts were therefore studied at both the whole heart and cellular level, assessing arrhythmic tendency and correlating this with electrophysiological parameters and structural changes.

## 2 Materials & methods

### 2.1 Experimental animals

This research has been regulated under the Animals (Scientific Procedures) Act 1986 Amendment Regulations 2012 following ethical review by the University of Cambridge Animal Welfare and Ethical Review Body (AWERB). Age-matched wild type (WT) and *Pgc-1β^−/−^* mice with an inbred C57/B6 genetic background were used for the study. *Pgc-1β^−/−^* mice were generated using a triple LoxP targeting vector as previously described (Lelliott et al., 2006). Mice aged between 3 and 4 months were classified as young, and aged mice were defined as those older than 12 months. Mice were housed in plastic cages within a temperature-controlled room maintained at 21 ± 1 °C and subjected to 12 h dark/light cycles. Sterile rodent chow (RM3 Maintenance Diet, SDS, Witham, Essex, UK) and water were available ad libitum.

### 2.2 Experimental solutions

All buffering media utilised in the study were based on Krebs-Henseleit (KH) solution, containing NaCl (119 mM), NaHCO_3_ (25 mM), KCl (4 mM), MgCl_2_ (1 mM), KH_2_PO_4_ (1.2 mM), CaCl_2_ (1.8 mM), glucose (10 mM) and sodium pyruvate (1.8 mM), pH adjusted to 7.4 and bubbled with 95% O_2_/5% CO_2_ (British Oxygen Company, Manchester, UK). All chemical reagents were purchased from Sigma-Aldrich (Dorset, Poole, UK) except where otherwise indicated. Hearts were electromechanically uncoupled using blebbistatin (20 μM, Selleckchem, Houston, USA) to minimize motion artifact during the microelectrode studies, permitting stable impalement of the cardiomyocyte.

### 2.3 Whole heart Langendorff preparation

Electrocardiograph and microelectrode studies were performed using a horizontal Langendorff perfusion system adapted for the murine heart incorporated into a Faraday cage, together with a light microscope (objective × 5, eyepiece × 5, W. Watson and Sons Limited, London, UK), custom built head stage and a warmed bath superfused with the buffering media. All equipment was electrically insulated. The stimulating and recording electrodes were positioned at appropriate positions on the right and left atrium respectively using two precision micromanipulators (Prior Scientific Instruments, Cambridge, UK).

Mice were anticoagulated with heparin sodium 200 IU (Sigma-Aldrich, Poole, UK) prior to sacrifice, administered into the intra-peritoneal space with a 27G hypodermic needle. Following an interval of 10 min, mice were killed by cervical dislocation (Schedule 1: UK Animals (Scientific Procedures) Act (1986)), a sternotomy and cardiectomy rapidly performed and the excised heart placed in ice-cold bicarbonate-buffered KH solution. The proximal segment of the aorta was identified and cannulated with a modified 21G hypodermic needle, and secured in place with an aneurysm clip (Harvard Apparatus, Kent, UK) and a 5-0 braided silk suture. The cannulated heart was mounted on to the Langendorff apparatus and retrogradely perfused with KH solution at a constant flow rate of 2.05 ml min^−1^ by a peristaltic pump (MINIPULS3, Gilson, Luton, UK) passing first through 200 μm and 5 μm Millipore filters (Millipore, Watford, UK) and maintained at 37 °C by a water jacket and circulator (model C-85A, Techne, Cambridge, UK). Upon perfusion, hearts were selected for experimentation if they demonstrated sustained intrinsic activity with a basic cycle length (BCL) <200 ms and 1:1 atrioventricular conduction (AV) for 10 min. Preparations meeting these criteria were subsequently perfused with 150 ml KH solution containing 20 μM blebbistatin and then normal KH solution throughout the remainder of the study.

### 2.4 Volume conductor electrocardiographic recordings

Whole heart volume conductor electrocardiographic (ECG) recordings were taken concurrently with intracellular recordings to distinguish between isolated cellular and generalised atrial phenomena. Two unipolar ECG leads were immersed into the warmed bath flanking the right and left atria respectively. Signals were amplified using a model NL104A amplifier (NeuroLog; Digitimer, Hertfordshire, UK), filtered at low and high cut-off frequencies of 5 and 500 Hz (model NL125/126 filter) and digitized using a model 1401 interface (Cambridge Electronic Design) for analysis with Spike II software (Cambridge Electronic Design).

### 2.5 Whole heart intracellular microelectrode recordings

Glass micropipettes were drawn from 1.2 mm outer diameter and 0.69 mm internal diameter borosilicate glass (Harvard Apparatus, Cambridge, UK) using a homebuilt microelectrode puller, and cut above the shoulders to an appropriate length. The microelectrodes were backfilled with 3 M KCl immediately before use, with tip resistances ranging between 15 and 25 MΩ. The filled microelectrode was mounted on to a right-angled microelectrode holder connected to a high-input impedance direct-current microelectrode amplifier system (University of Cambridge, Cambridge, UK). Intracellular voltage was measured relative to that of the Ag/AgCl reference electrode. AP recordings were used for analysis if obtained from an impalement associated with the abrupt appearance of a resting membrane potential (RMP) between −65 mV and −90 mV, stable and normal AP morphology and an AP amplitude >75 mV.

Hearts were placed in the anatomical position within the bath with the left atrium (LA) reflected back and fixed in position using three A1 insect pins. A bipolar platinum-coated stimulating electrode (NuMed, New York, USA) was positioned against the epicardial surface of the right atrium, pacing the heart using square-wave stimuli of 2 ms duration using a constant voltage stimulator (model DS2A-Mk.II, Digitimer, Welwyn Garden City, Herts., UK) controlled by Spike II software (Cambridge Electrical Design, Cambridge, UK) and delivering a voltage that was twice the diastolic excitation threshold plus 0.5 mV. The initial experiments examined the hearts under conditions of regular pacing at a basic cycle length (BCL) of 125 ms (8 Hz). Hearts were then studied using a programmed electrical stimulation (PES) protocol comprising drive trains of eight regularly paced (S1) beats at a BCL of 125 ms, followed by an isolated premature extra stimulus (S2) every ninth beat. The S2 stimulus was imposed at progressively shortening S1-S2 coupling intervals, initially being 89 ms and reducing by 1 ms every subsequent cycle to a final coupling interval of 5 ms. The protocol was terminated upon establishment of the atrial effective refractory period (ERP), defined as the first S1-S2 coupling interval at which the S2 stimulus failed to successfully elicit an AP, or sustained arrhythmia was observed.

### 2.6 Quantification of AP parameters and arrhythmic incidence

**Fig. 1:**
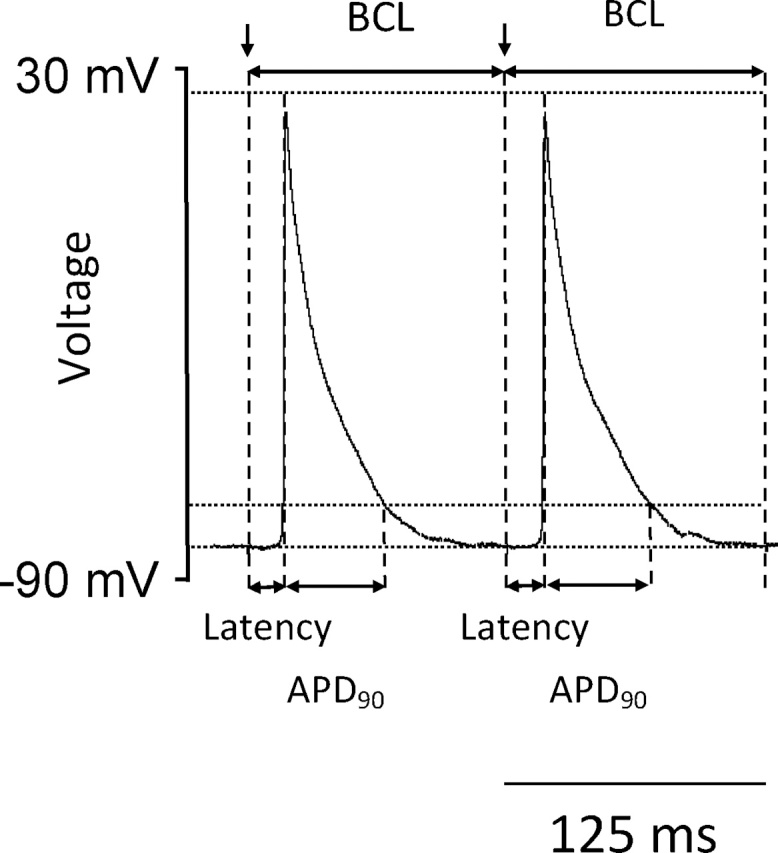
Basic measures of atrial action potential (AP) propagation, activation and recovery. AP amplitude was measured from the baseline to the peak voltage excursion. AP duration was measured as 90% recovery to baseline (APD_90_), and AP latencies were measured as the time elapsed between the pacing stimulus and peak of the AP. Maximum rates of depolarization (d*V*/d*t*)_max_ were calculated from the first differential of the intracellular AP waveform.

The electrophysiological parameters were calculated from each AP individually and averaged across the protocol to give an overall mean for each heart. The AP amplitude was measured from the baseline to the peak voltage excursion and the AP duration was measured as 90% recovery to baseline (APD_90_) (Fig. 1). AP latencies were measured as the time elapsed between the pacing stimulus and peak of the AP. Maximum rates of AP depolarization (d*V*/d*t*)_max_ were calculated from the differentiated intracellular AP waveform. The incidence of abnormal atrial rhythms were determined from the regular pacing and PES protocols, correlating cellular phenomena with tissue-level activity. Isolated non-triggered APs were classified as ectopic beats, two successive non-triggered beats termed a couplet and atrial tachycardia (AT) was defined as an episode consisting of ≥3 consecutive non-triggered beats.

### 2.7 Quantification of cardiac fibrosis

The quantification of cardiac fibrosis was performed as previously described (Jeevaratnam et al., 2011). Briefly, the excised heart was first flushed with KH solution and then perfused for five minutes with 4% buffered paraformaldehyde before being immersed in the paraformaldehyde overnight. Following the fixation process, longitudinal cardiac sections were cut and subjected to routine tissue processing and paraffin embedding. Serial sections of 7 μm thickness were then taken and stained with picrosirius red for fibrotic change. All sections were subsequently viewed, magnified and digitally acquired using the Nano Zoomer 2.0 Digital Pathology system (Hamamatsu, Hertfordshire, UK). A custom made 17 cm × 30 cm morphometric grid was superimposed on each magnified photomicrograph and each successive 1 cm × 1 cm square, corresponding to 0.2 mm × 0.2 mm area of tissue, was scored first for the presence or absence of cardiac tissue, and in turn for presence of fibrosis. The degree of fibrosis was quantified as the proportion of squares occupied by cardiac tissue showing evidence of fibrotic change. The analysis was performed independently by two investigators blinded to the animal genotype and age, and an inter-class correlation coefficient analysis (ICC) was performed to assess the consistency of their results, which can be interpreted as follows: 0–0.2 indicates *poor* agreement; 0.3–0.4 indicates *fair* agreement; 0.5–0.6 indicates *moderate* agreement; 0.7–0.8 indicates *strong* agreement; and >0.8 indicates *almost perfect* agreement.

### 2.8 Statistical analysis

Data from AP recordings were analysed using a custom written programme in the python programming language and all statistical analysis performed in the R programming language (R Core Team, 2015). Discrete incidences of abnormal rhythms were separated according to their type and the pacing protocol, either regular pacing or PES, in which they were observed. As often multiple episodes of AT were seen during a single protocol, the propensity to arrhythmia was expressed as protocols with one or more episodes of AT expressed relative to the total number studied, and compared using the Fisher Exact Test. Parameters describing electrophysiological properties measured during regular pacing were compared using a two-way analysis of variance (ANOVA) testing for significant effects of genotype, ageing, and an interaction between the two. Where the *F*-ratio yielded a significant result, post-hoc Tukey honestly significant testing was performed. Similar electrophysiological measurements from PES protocols were compared in two separate ways. Firstly, differences spanning the duration of the protocol were compared using ANOVA analysis of area under the curve (AUC) values for each group. To further evaluate the temporal nature of any differences between groups, mean protocol start and protocol end values for each experimental group were compared in the same manner as data from the regular pacing protocol. Where the data from PES protocols was normalised, the corresponding data from regular pacing was used as reference values. Data are expressed as mean ± standard error of the mean (SEM), and in all cases a p < 0.05 was taken to be significant, with application of Bonferroni correction where appropriate.

## 3 Results

The experiments evaluated the influence of ageing and mitochondrial dysfunction, through homozygous deficiency in *Pgc-1β,* upon atrial arrhythmic tendency and the associated electrophysiological alterations. Simultaneous ECG and intracellular microelectrode readings were recorded from Langendorff perfused WT and *Pgc-1β*^−/−^ hearts and the presence or otherwise of an arrhythmic substrate was then correlated with structural changes at the organ level.

### 3.1 Pgc-1β^−/−^ hearts develop an age-related arrhythmic phenotype

**Fig. 2:**
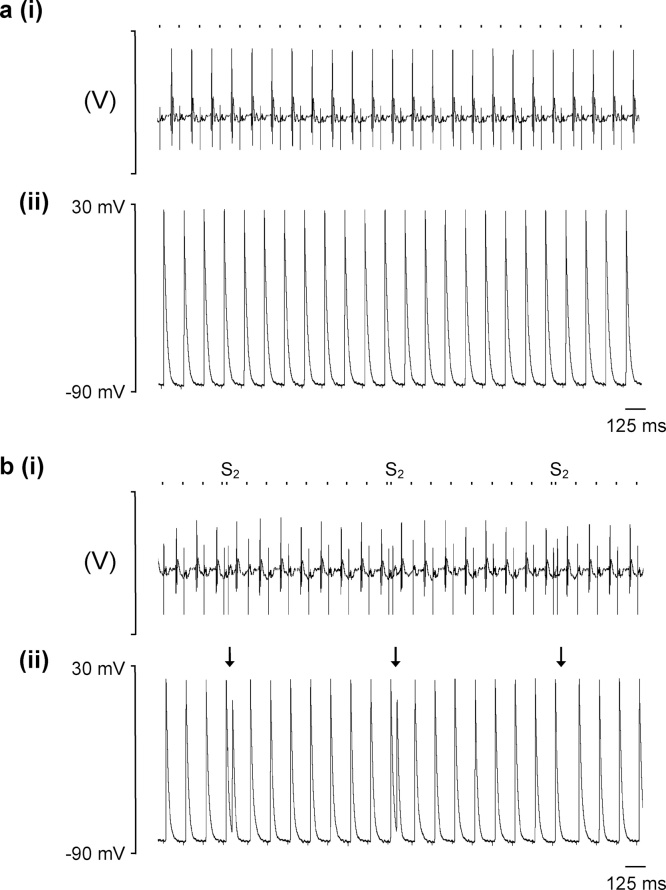
Typical recordings from Langendorff-perfused WT hearts during regular pacing and programmed electrical stimulation. Electrocardiograph (ECG) (i) and left atrial intracellular action potential (AP) recordings (ii) during (a) regular 8 Hz pacing and (b) a protocol imposing programmed electrical stimulation with a refractory outcome. The timings of stimulus delivery are given by the dashed bar above the AP recordings, and corresponding stimulation artefacts can be seen on the ECG and AP traces, preceding the respective complexes. In panel (B), arrows indicate the imposition of S2 extrastimuli. The first two S2 stimuli trigger APs, whereas the third S2 stimulus fails to elicit a response, thus representing a refractory outcome.

Volume conductor ECGs and intracellular action potential (AP) recordings were first obtained from Langendorff perfused hearts during regular pacing at a BCL of 125 ms (8 Hz) mimicking murine resting heart rates, thus enabling quantification of occurrence of spontaneous arrhythmia and electrophysiological characterisation under conditions of baseline activity. Fig. 2(a(i)) demonstrates a typical ECG recording from a young WT heart during regular 8 Hz pacing and Fig. 2(a(ii)) is the simultaneous intracellular AP from a left atrial (LA) cardiomyocyte. The intracellular recordings confirmed normally polarised resting membrane potentials (RMPs) statistically indistinguishable between groups (young WT: −76.62 ± 1.37 mV, n = 26; aged WT: −76.72 ± 1.47 mV, n = 27, young *Pgc-1β*^−/−^: -75.82 ± 0.68 mV, n = 34; aged *Pgc-1β*^−/−^: −77.43 ± 1.49 mV, n = 25). Similarly, AP amplitudes confirmed positive AP overshoots through all experimental groups, consistent with intracellular recordings from viable atrial cardiomyocytes in situ, though AP amplitudes were marginally lower in aged *Pgc-1β*^−/−^ hearts (young WT: 96.76 ± 1.14 mV, n = 26; aged WT: 96.92 ± 1.55 mV, n = 27, young *Pgc-1β*^−/−^: 93.19 ± 1.12 mV, n = 34; aged *Pgc-1β*^−/−^: 91.21 ± 1.63 mV, n = 25, p < 0.05). No spontaneous arrhythmias were observed from hearts in any experimental group during the regular pacing protocols.

**Fig. 3:**
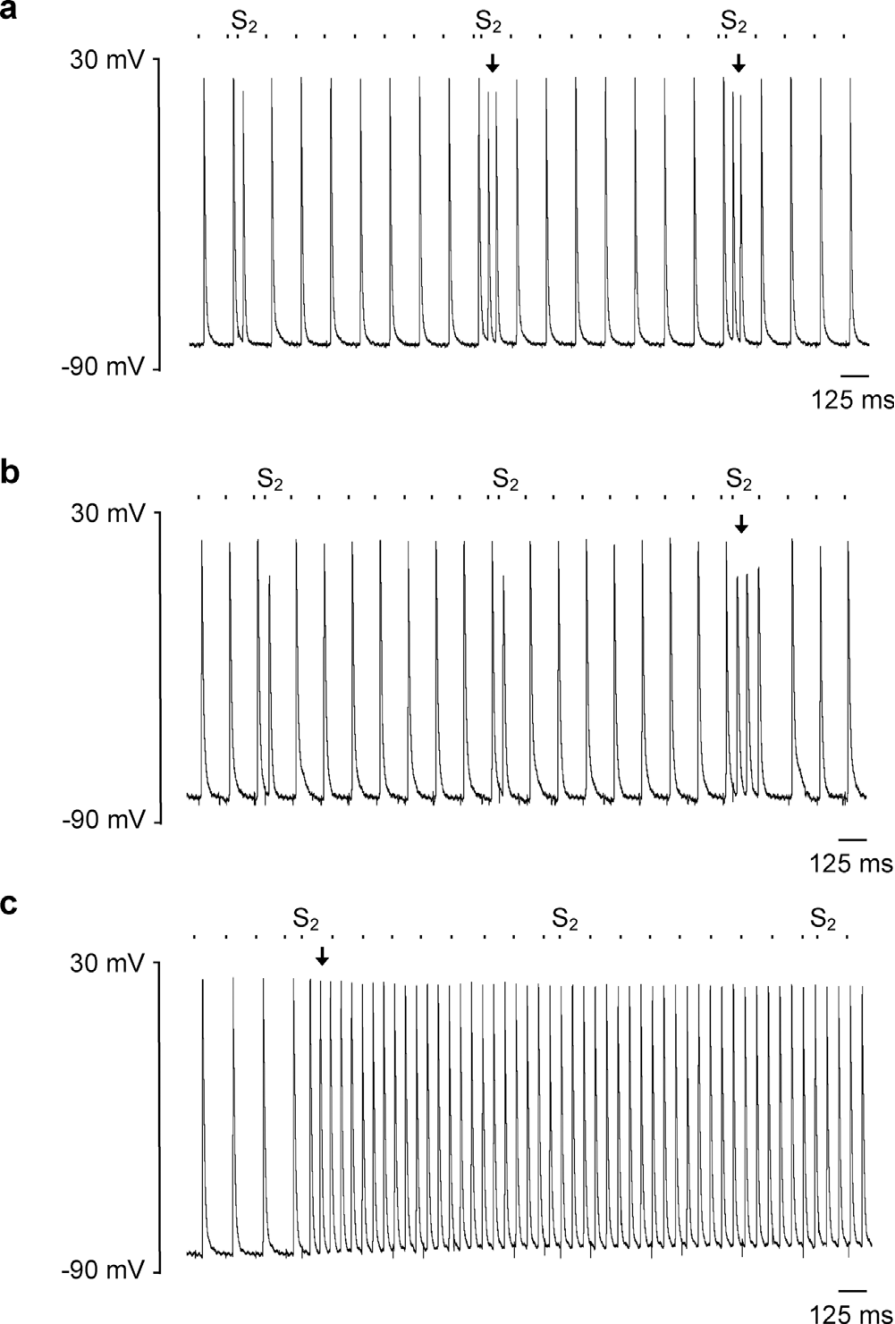
Examples of intracellular records of abnormal rhythms elicited by S2 premature stimuli during PES pacing, including (a) isolated ectopic beats, (b) paired beats forming couplets and (c) episodes of atrial tachycardia (AT) defined as three or more consecutive non-stimulated beats. The timings of stimulus delivery are given by the dashed bar above the AP recordings and arrows indicate the onset of the abnormal rhythm.

Hearts were then subjected to a programmed electrical stimulation (PES) protocol consisting of repeated cycles of nine beats, of which the first eight (S1) beats were separated by a regular interval of 125 ms and the ninth was a premature extra stimulus (S2) at an initial S1-S2 coupling interval of 89 ms that was decremented by 1 ms with each successive cycle. This permitted evaluation of the arrhythmic tendency of hearts in response to provocation with an imposed premature S2 beat, the alterations in electrophysiological parameters with varying coupling intervals, and differences in atrial effective refractory periods (ERPs) between groups. Fig. 2(b) shows typical (i) ECG and (ii) AP recordings during PES pacing from a young WT heart with a refractory as opposed to an arrhythmic outcome. Several abnormal rhythms were observed during PES pacing as exemplified in Fig. 3. These were triggered by the S2 premature stimulus and included isolated ectopic beats (Fig. 3(a)), paired beats termed a couplet (Fig. 3(b)) and episodes of atrial tachycardia (AT) defined as three or more consecutive non-stimulated beats (Fig. 3(c)).

Table 1 summarises the number of episodes of the different abnormal rhythms observed during PES, stratified by experimental group. Incidences of atrial tachycardia (AT) were significantly greater in aged *Pgc-1β*^−/−^ hearts compared to any other group with respect to the overall proportion affected (p < 0.05, Fisher Exact Test) and the number of arrhythmic events per individual heart, suggesting an arrhythmic phenotype associated with mitochondrial dysfunction that progresses with age. AT was more frequently observed in the aged *Pgc-1β*^−/−^ group compared to either of the WT groups, with no difference in incidence observed between the young and aged WT groups. Although a similar proportion of young and aged WT hearts were arrhythmic, arrhythmic event rates were higher in the latter further reinforcing an effect of age upon arrhythmic risk. Thus ANOVA of mean AT events per heart demonstrated a significant effect of genotype (*F* = 7.13, p < 0.01) with *Pgc-1β*^−/−^ hearts having higher event rates and age (*F* = 7.26, p < 0.01), but no interactive effect (*F* = 2.37, p > 0.05). Post hoc Tukey tests demonstrated significant differences in rates of AT between aged *Pgc-1β*^−/−^ hearts and young WT hearts (p < 0.01), aged WT hearts (p < 0.05) and young *Pgc-1β*^−/−^ hearts (p < 0.05).

**Table 1:** Summary of arrhythmic events during programmed electrical stimulation.

Experimental group	No. that developed AT (n/total)	Ectopic beats (mean ± SEM)	Couplets (mean ± SEM)	AT (mean ± SEM)	Critical coupling interval (mean ± SEM)
Young Wild Type	5/27	1.48 ± 0.50	0.41 ± 0.26	0.26^**^ ± 0.11	28.71 ^#^ ± 3.46
Aged Wild Type	4/27	0.79 ± 0.53	1.17 ± 0.62	0.48^†^ ± 0.36	32.71 ± 4.21
Young *Pgc-1β^−/−^*	11/34	1.22 ± 0.47	0.42 ± 0.21	0.86^‡^ ± 0.35	35.70 ± 1.35
Aged *Pgc-1β^−/−^*	12/25*	1.64 ± 0.66	1.28 ± 0.55	2.64^**,†,‡^ ± 0.70	39.39 ^#^ ± 1.12

Symbols denote significant difference based on post hoc analysis, performed if the *F* value from two-way ANOVA was significant. Single, double and triple symbols denote p < 0.05, p < 0.01 and p < 0.001 respectively.

**Fig. 4:**
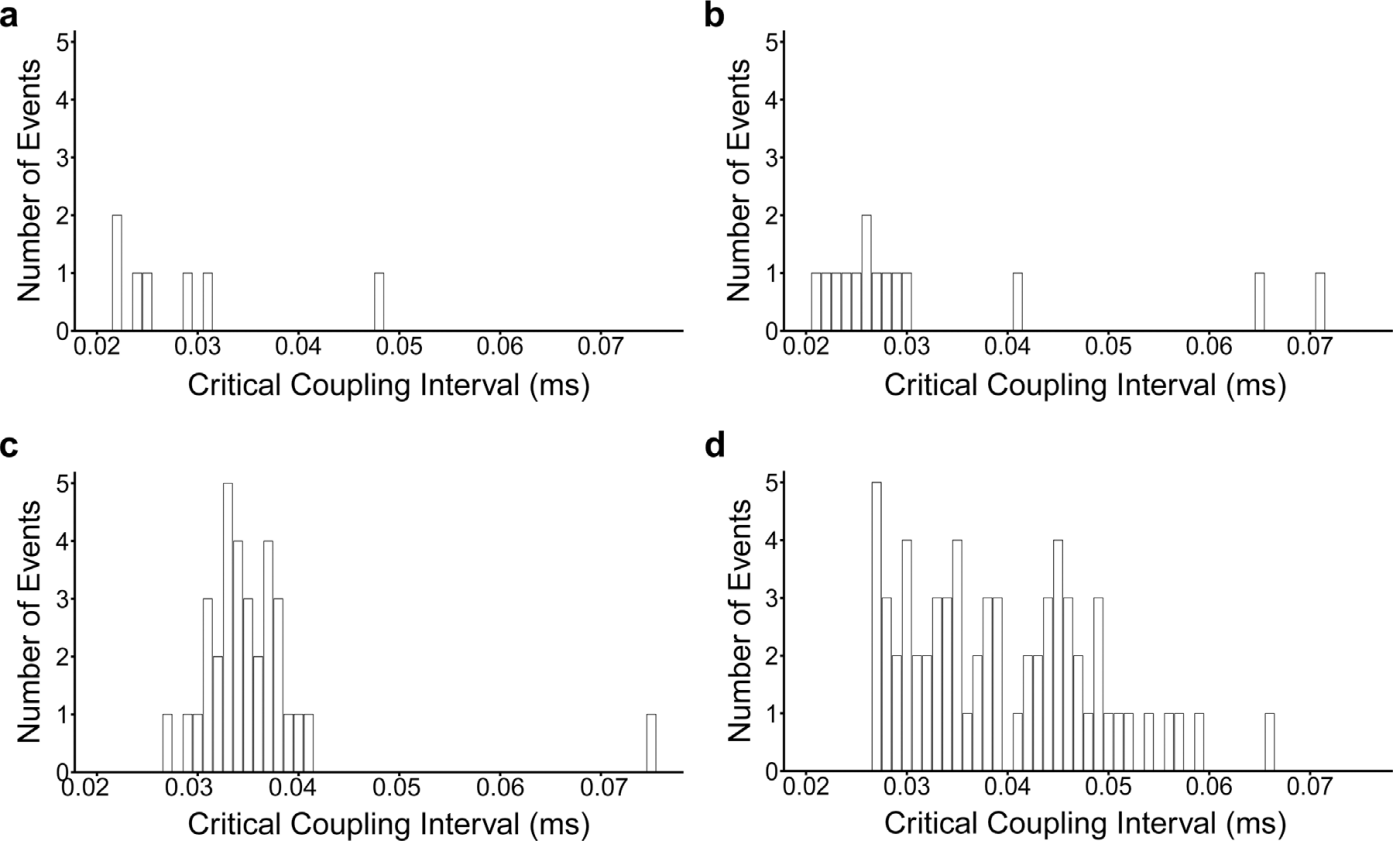
Stratification of the occurrence of AT episodes by critical coupling intervals in young ((a), (c)) and old ((b), (d)), WT ((a), (b)) and *Pgc-1β*^−/−^ hearts ((c), (d)). *Pgc-1β*^−/−^ hearts display vulnerability to arrhythmia earlier, and through wider range of S1-S2 coupling intervals.

This increased propensity to AT is further highlighted on analysis of the critical coupling intervals, given by the S1-S2 coupling interval at which arrhythmia was triggered as shown in Fig. 4. Episodes of AT in WT hearts (Fig. 4(a) and (b)) were triggered predominantly at latter parts of the PES protocol corresponding to shorter S1-S2 coupling intervals. In contrast *Pgc-1β*^−/−^ hearts developed arrhythmias at earlier stages of the protocol and over a wider range of coupling intervals (Fig. 4(c) and (d)), with aged *Pgc-1β*^−/−^ hearts particularly appearing vulnerable throughout the duration of the protocol. The mean critical coupling interval was longer in *Pgc-1β*^−/−^ hearts (*F* = 8.35, p  < 0.01) and aged hearts (*F* = 3.93, p < 0.05), though no interactive effect was observed (*F* = 0.004, p > 0.05). Post hoc analysis demonstrated significant differences between aged *Pgc-1β*^−/−^ and young WT hearts (p < 0.05) and a trend to significance between aged *Pgc-1β*^−/−^ and aged WT hearts (p < 0.10).

### 3.2 Action potential parameters during regular pacing

These differing arrhythmic profiles were next compared with electrophysiological parameters corresponding to AP initiation, propagation and recovery during regular pacing at 8 Hz. AP initiation was measured first through maximum rates of AP depolarisation (d*V*/d*t*)_max_, derived from left atrial intracellular microelectrode recordings. (d*V*/d*t*)_max_ measurements quantify the depolarisation of the cardiomyocyte membrane capacitance by regenerative inward Na^+^ current. Reductions in (d*V*/d*t*)_max_ are known to correlate with compromised conduction velocity of an AP wavefront, potentially providing a substrate permissive to AP re-entry and arrhythmia. *Pgc-1β^−/−^* hearts showed significantly lower values of (d*V*/d*t*)_max_ compared to WT (*F* = 18.41, p < 0.001) but there was no effect of age (*F* = 0.17, p > 0.05) or interaction between age and genotype (*F* = 0.001, p > 0.05). On post hoc Tukey testing, each *Pgc-1β^−/−^* group, whether young or aged, showed significantly lower (d*V*/d*t*)_max_ values than either of the WT groups (Table 2).

**Table 2:** Action potential properties in WT and Pgc-1β-/- hearts during regular 8 Hz pacing.

Experimental group	(dV/dt)_max_ (V s^−1^)	AP latency (ms)	APD_90_ duration (ms)	Effective refractory period (ms)	Wavelength
Young Wild Type	171.06*, † ± 6.60	17.69***, †††, ‡‡ ± 0.23	24.87 ± 1.18	26.62 ± 1.27	4.21*, † ± 0.25
Aged Wild Type	171.73‡‡, # ± 5.15	23.93*** ± 1.24	25.51 ± 1.59	27.33 ± 1.77	4.34‡‡, ## ± 0.29
Young Pgc-1β-/-	141.28*, ‡‡ ± 7.19	24.61††† ± 0.65	23.97 ± 0.93	29.82 ± 0.54	3.28*, ‡‡ ± 0.16
Aged Pgc-1β-/-	142.39†, # ± 8.08	23.48‡‡ ± 1.66	23.28 ± 2.16	28.33 ± 1.33	3.15†, ## ± 0.25

All values are given as mean (±SEM).

Symbols denote significant difference based on post hoc analysis, performed if the F value from two-way ANOVA was significant. Single, double and triple symbols denote p < 0.05, p < 0.01 and p < 0.001 respectively.

AP conduction through respective cardiac chambers is determined not only by the properties of the inward Na^+^ current, reflected in the cellular (d*V*/d*t*)_max_ values, but also the total membrane capacitance and resistance (King et al., 2013a). AP propagation was therefore further assessed through comparisons of AP latency times, measured as the time intervening between stimulus delivery at the right atrial pacing site and the peak AP voltage measured at the left atrial (LA) recording site. In all experiments, the stimulating electrode was consistently positioned at the posterior aspect of the RA and recordings were made from the central region of the LA, minimising variability in distances between the respective electrodes. ANOVA of AP latency times demonstrated significant effects of genotype (*F* = 9.91, p < 0.01), age (*F* = 5.32, p < 0.05) and an interaction of the two (*F* = 12.47, p < 0.001). As detailed in Table 2, these differences were driven by young WT hearts, which had significantly shorter AP latency times than aged WT hearts (p < 0.001), young *Pgc-1β^−/−^* hearts (p < 0.001), and aged *Pgc-1β^−/−^* hearts (p < 0.01), There was no significant difference between the aged WT hearts and either of the *Pgc-1*β*^−/−^* groups.

Pro-arrhythmic tissue substrate has also been associated with altered repolarisation associated with action potential shortening or prolongation (Killeen et al., 2007, Sabir et al., 2007a, Sabir et al., 2007b). Electrophysiological parameters describing repolarisation are given in Table 2. No differences in APD at 90% repolarisation (APD_90_) were found during regular 8 Hz pacing either through effects of genotype (*F* = 1.07, p > 0.05), age (*F* = 0.001, p > 0.05) or an interaction of the two (*F* = 0.20, p > 0.05). Repolarisation properties were further assessed through measurement of the effective refractory period (ERP) obtained from the PES protocol, defined as the longest S1-S2 coupling interval at which the S2 stimulus failed to trigger an AP. The ERP generally correlates with the APD, and in keeping with this no differences between groups were observed when compared according to genotype (*F* = 3.36, p > 0.05) or age (*F* = 0.04, p > 0.05).

### 3.3 Action potential parameters following premature extrasystolic stimuli

The trigger-substrate model of arrhythmogenesis involves initiation of the abnormal rhythm through an arrhythmogenic trigger, such as an extrasystole, occurring within a pro-arrhythmic substrate capable of sustaining the arrhythmia (Antzelevich et al., 1999, Kalin et al., 2010). The PES protocol consisted of pulse trains of S1 beats 125 ms apart, punctuated every ninth beat by a premature S2 stimulus, mimicking such triggering extrasystoles. The external application of these premature beats thus controlled for incidence of ectopic stimuli between groups and so assessed for the presence of such an arrhythmogenic substrate.

**Fig. 5:**
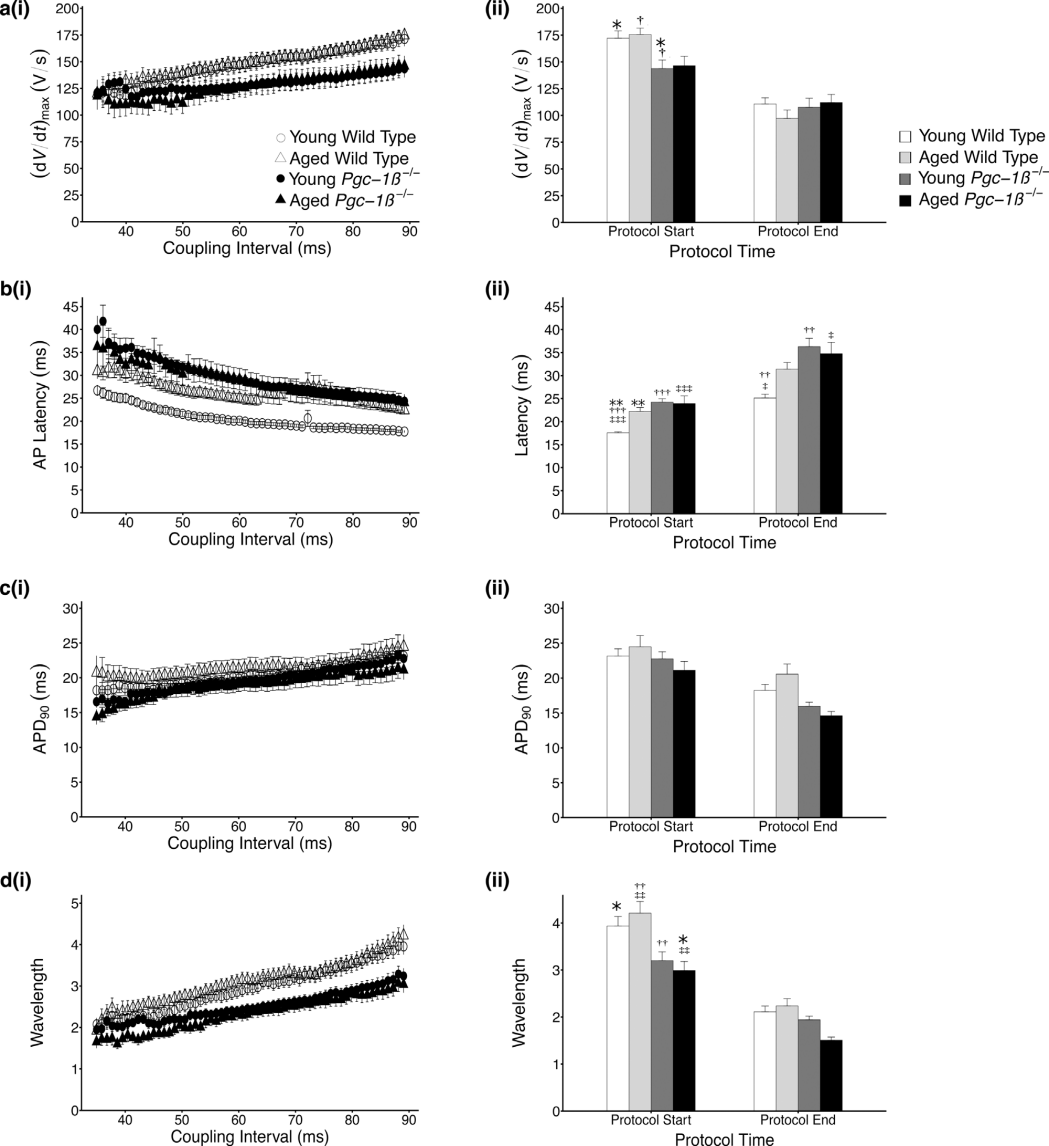
Plots of mean ± SEM (a) (d*V*/d*t*)_max_, (b) AP latency and (c) time to 90% AP recovery (APD_90_) and (d) AP wavelength for APs obtained in response to S2 stimuli (i) through the range of coupling intervals explored, reducing from 89 ms to 30 ms. Panel (ii) for each provides a comparison of these values at the beginning and termination of the pacing protocol, corresponding to a refractory outcome or the onset of sustained arrhythmia. The symbols denote significant differences between each pair, obtained from post hoc Tukey testing, which was conducted if the ANOVA indicated a significant outcome. Single, double and triple symbols denote p < 0.05, p < 0.01 and p < 0.001 respectively.

Fig. 5(a(i)) plots mean (d*V*/d*t*)_max_ values for APs triggered by S2 stimuli across the range of coupling intervals explored during the PES protocol. All experimental groups displayed the expected progressively reduced (d*V*/d*t*)_max_ values with shortening of the S1-S2 coupling interval. In common with findings obtained during regular pacing, an analysis of the areas beneath the data curve (AUC) demonstrated that the overall rates of depolarisation were significantly higher in WT than *Pgc-1β^−/−^* hearts (*F* = 6.41, p < 0.05) (Table 3); there were no effects of age (*F* = 0.84, p > 0.05) or interacting effects of genotype and age (*F* = 0.27, p > 0.05). The difference between WT and *Pgc-1β^−/−^* hearts was most pronounced at the beginning of the protocol (Fig. 5(a(ii))) (ANOVA − genotype: *F* = 13.19, p < 0.001; age: *F* = 0.15, p > 0.05; interaction: *F* = 0.002, p > 0.05) and was of a similar magnitude as had been observed during 8 Hz pacing. On post hoc analysis, each WT group showed significantly higher (d*V*/d*t*)_max_ values compared against either of the *Pgc-1β^−/−^* groups. In contrast, no difference in (d*V*/d*t*)_max_ values was observed at the shortest coupling intervals at the end of the protocol, whether tested for effects of genotype (*F* = 0.09, p > 0.05), age (*F* = 0.18, p > 0.05) or interaction between these factors (*F* = 0.31, p > 0.05).

**Table 3:** Area under the curve analysis for S2 triggered APs during programmed electrical stimulation.

Experimental group	(dV/dt)_max_ (V × 10^−3^)	AP latency (ms^2^)	APD_90_ (ms^2^)	Wavelength (ms)
Young Wild Type	6.68 ± 0.32	1.06††, ‡ ± 0.04	1.06 ± 0.05	0.085 ± 0.005
Aged Wild Type	6.11 ± 0.35	1.21 ± 0.05	0.93 ± 0.06	0.082 ± 0.007
Young Pgc-1β-/-	5.44 ± 0.37	1.39†† ± 0.08	0.95 ± 0.05	0.066 ± 0.005
Aged Pgc-1β-/-	5.30 ± 0.64	1.38‡ ± 0.01	0.82 ± 0.06	0.065 ± 0.005

All values are given as mean (±SEM).

Symbols denote significant difference based on post hoc analysis, performed if the F value from two-way ANOVA was significant. Single, double and triple symbols denote p < 0.05, p < 0.01 and p < 0.001 respectively.

Similar plots for AP latencies are given in Fig. 5(b(i)) and 5(b(ii)). In keeping with findings during regular 8 Hz pacing and the observed differences in (d*V*/d*t*)_max_ values, AP latency times were significantly prolonged in *Pgc-1β^−/−^* hearts when surveyed through the entirety of the protocol on AUC analyses (*F* = 12.98, p < 0.001), but there were no independent effects of age (*F* = 1.11, p > 0.05) or compound effects of age and genotype (*F* = 1.36, p > 0.05). Young WT hearts had significantly shorter AP latency times compared to young *Pgc-1β^−/−^* hearts (p < 0.01) and aged *Pgc-1β^−/−^* hearts (p < 0.05) on post hoc testing. ANOVA of AP latencies at the longest S1-S2 intervals demonstrated significant effects of genotype (*F* = 19.23, p < 0.001), age (*F* = 4.79, p < 0.05) and interacting effects of genotype and age (*F* = 6.12, p < 0.05). Here the AP latency times for young WT hearts were significantly shorter than all other groups including aged WT (p < 0.01), young *Pgc-1β^−/−^* (p < 0.001) and aged *Pgc-1β^−/−^* hearts (p < 0.001). AP latencies progressively lengthened in all groups as the S1-S2 interval shortened but to varying degrees. Thus, at the shortest S1-S2 intervals, a significant difference between WT and *Pgc-1β^−/−^* hearts persisted (*F* = 10.15, p < 0.001); however significant effects of age (*F* = 1.19, p > 0.05) or interaction (*F* = 2.79, p > 0.05) were no longer evident. AP latency times remained significantly shorter in young WT hearts when compared with young *Pgc-1β^−/−^* (p < 0.01) and aged *Pgc-1β^−/−^* hearts (p < 0.05); however the lengthening of AP latency in aged WT was less pronounced than that of the *Pgc-1β^−/−^* hearts and thus the difference with the young WT hearts was no longer significant.

The adaptation of AP duration, given by APD_90_ values, through progressively shortening S1–S2 coupling intervals is shown in Fig. 5(c(i)). Overall APD_90_ times did not differ between experimental groups (ANOVA – genotype: *F* = 2.95, p > 0.05; age: *F* = 1.71, p > 0.05; interaction: *F* = 0.002, p > 0.05), reflecting the findings during regular 8 Hz pacing. Accordingly, APD_90_ times at the beginning of the protocol, corresponding to the longest S1-S2 intervals, also did not differ between groups (ANOVA – genotype: *F* = 1.63, p > 0.05; age: *F* = 0.02, p > 0.05; interaction: *F* = 1.30, p > 0.05). APD_90_ times in all groups displayed the expected shortening as the S1-S2 interval decreased; however a small but significant difference in APD_90_ between WT and *Pgc-1β^−/−^* hearts was seen at the shortest coupling intervals (*F* = 6.60, p < 0.05), where *Pgc-1β^−/−^* hearts had shorter APD_90_. No differences were noted based upon age (*F* = 0.02, p > 0.05), or interacting effects of genotype and age (*F* = 1.52, p > 0.05). There were no significant differences between groups on post hoc Tukey testing.

Reductions in the AP wavelength have been suggested to correlate with increased arrhythmic risk, indicating the presence of substrate favorable to AP re-entry (Davidenko et al., 1995, Krogh-Madsen et al., 2012, Pandit and Jalife, 2013, Weiss et al., 2005). It has previously been calculated from terms relating to AP conduction and AP duration (Matthews et al., 2013), and similar analyses were conducted in the present study. *Pgc-1β^−/−^* hearts had significantly shorter wavelength values at resting hearts as measured during 8 Hz pacing (*F* = 20.62, p < 0.001), however there were no effects of ageing (*F* = 0.01, p > 0.05) or interacting effects of the two variables (*F* = 0.32, p > 0.05) (Table 2). AP wavelength profiles for beats triggered by S2 extrastimuli during PES pacing are shown in Fig. 5(d). Wavelengths were shorter throughout the protocol in *Pgc-1β^−/−^* hearts (*F* = 9.19, p < 0.01), with no independent effect of age (*F* = 0.03, p > 0.05) or interacting effects of genotype and age (*F* = 0.01, p > 0.05). The differences between WT and *Pgc-1β^−/−^* hearts noted during regular 8 Hz pacing was similarly evident at the longest S1-S2 intervals (*F* *=* 18.93, p < 0.01) with no other significances noted (ageing: *F* *=* 0.01, p > 0.05; genotype – age interaction: *F* *=* 1.20, p > 0.05) (Fig. 5(d(ii))). Post hoc Tukey tests revealed significant differences between young WT and aged *Pgc-1β^−/−^* hearts (p < 0.05), aged WT and young *Pgc-1β^−/−^* hearts (p < 0.01), and aged WT and aged *Pgc-1β^−/−^* hearts (p < 0.01). Wavelength values were reduced in all groups as the S1-S2 coupling interval shortened, correlating with the increased vulnerability to arrhythmias seen in all hearts. However, AP wavelengths for young *Pgc-1β^−/−^* hearts more closely converged to those of both WT groups, whereas wavelengths remained shorter in aged *Pgc-1β^−/−^* hearts. Thus though *Pgc-1β^−/−^* hearts continued to display significantly shorter wavelengths at the shortest S1-S2 intervals (ANOVA – genotype: *F* *=* 5.00, p < 0.05; age: *F* *=* 0.51, p > 0.05, interaction: *F* = 2.40, p > 0.05), no significant differences were noted between any groups on post hoc testing.

### 3.4 Relative changes in action potential parameters following premature extrasystolic stimuli

**Fig. 6:**
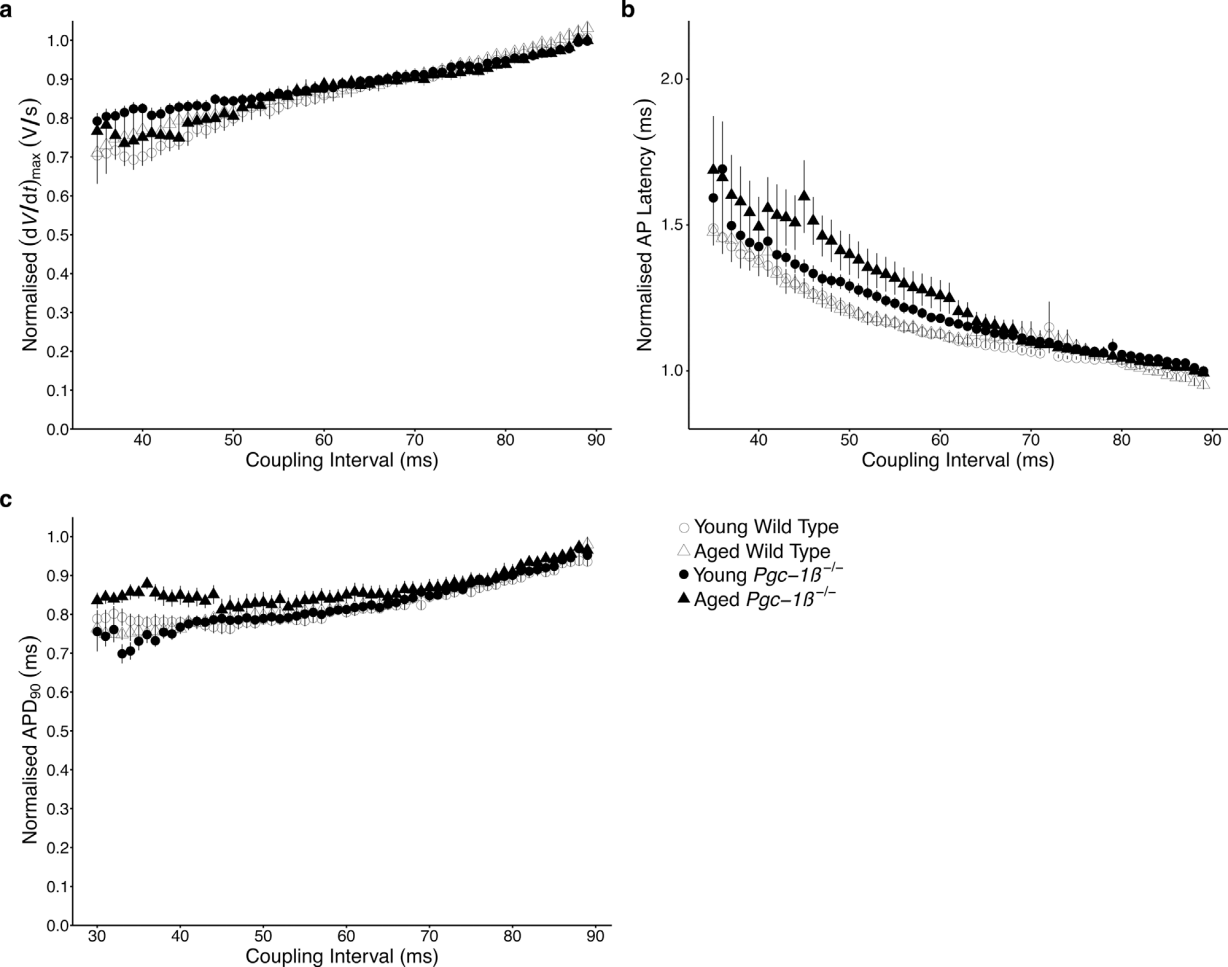
Plots of mean ± SEM (a) (d*V*/d*t*)_max_, (b) AP latency, (c) time to 90% AP recovery (APD_90_) in APs obtained in response to S2 stimuli, normalized to their corresponding values obtained during regular 8 Hz pacing through progressively shortening S1-S2 coupling intervals.

The energetic dysfunction associated with mitochondrial impairment would be expected to particularly compromise cardiac activity in the stressed state. Indeed *Pgc-1α* deficient hearts show normal contractile function at baseline but develop pronounced cardiac failure in response to aortic banding. *Pgc-1β^−/−^* hearts are known to develop chronotropic incompetence in response to adrenergic challenge despite normal resting heart rates. To further characterise the cardiac phenotype in response to increasing metabolic demand in the form of shortening pacing intervals, electrophysiological parameters during PES pacing were normalised internally to their corresponding values measured during regular 8 Hz pacing. The normalised profiles for the relevant parameters are shown in Fig. 6(a–c). Normalised (d*V*/d*t*)_max_ (Fig. 6a) and normalised APD_90_ values (Fig. 6c) displayed similar reductions with shortening S1-S2 intervals, and there were no significant differences in AUC values for either parameter (Table 4). Despite differing absolute AP latency times, normalised AP latency profiles for young and aged WT hearts were similar (Fig. 6(b)). In contrast *Pgc-1β^−/−^* hearts displayed greater increments in normalised latency with shortening S1-S2 intervals, with aged *Pgc-1β^−/−^* hearts appearing most compromised. ANOVA analysis of AUC values for normalised AP latency showed no independent effects of genotype (*F* = 0.49, p > 0.05) or age (*F* = 0.05, p > 0.05), but a significant interacting effect of the two (*F* = 4.31, p < 0.05) with aged *Pgc-1β^−/−^* hearts having the highest AUC values. No significant differences were seen on individual comparisons with post hoc Tukey testing.

**Table 4:** Area under the curve analysis for S2 triggered APs during programmed electrical stimulation, normalised to corresponding values from regular pacing.

Experimental group	(dV/dt)_max_ (V × 10^−3^)	AP latency (ms^2^)	APD_90_ (ms^2^)
Young Wild Type	0.045 ± 0.001	0.039 ± 0.02	0.025 ± 0.001
Aged Wild Type	0.041 ± 0.002	0.035 ± 0.002	0.023 ± 0.002
Young Pgc-1β-/-	0.042 ± 0.002	0.037 ± 0.002	0.023 ± 0.001
Aged Pgc-1β-/-	0.041 ± 0.003	0.043 ± 0.003	0.023 ± 0.002

All values are given as mean (±SEM).

Symbols denote significant difference based on post hoc analysis, performed if the F value from two-way ANOVA was significant. Single, double and triple symbols denote p < 0.05, p < 0.01 and p < 0.001 respectively.

### 3.5 Contrasting impacts of (dV/dt)_max_ upon AP latency in WT and Pgc-1β^−/−^ hearts

**Fig. 7:**
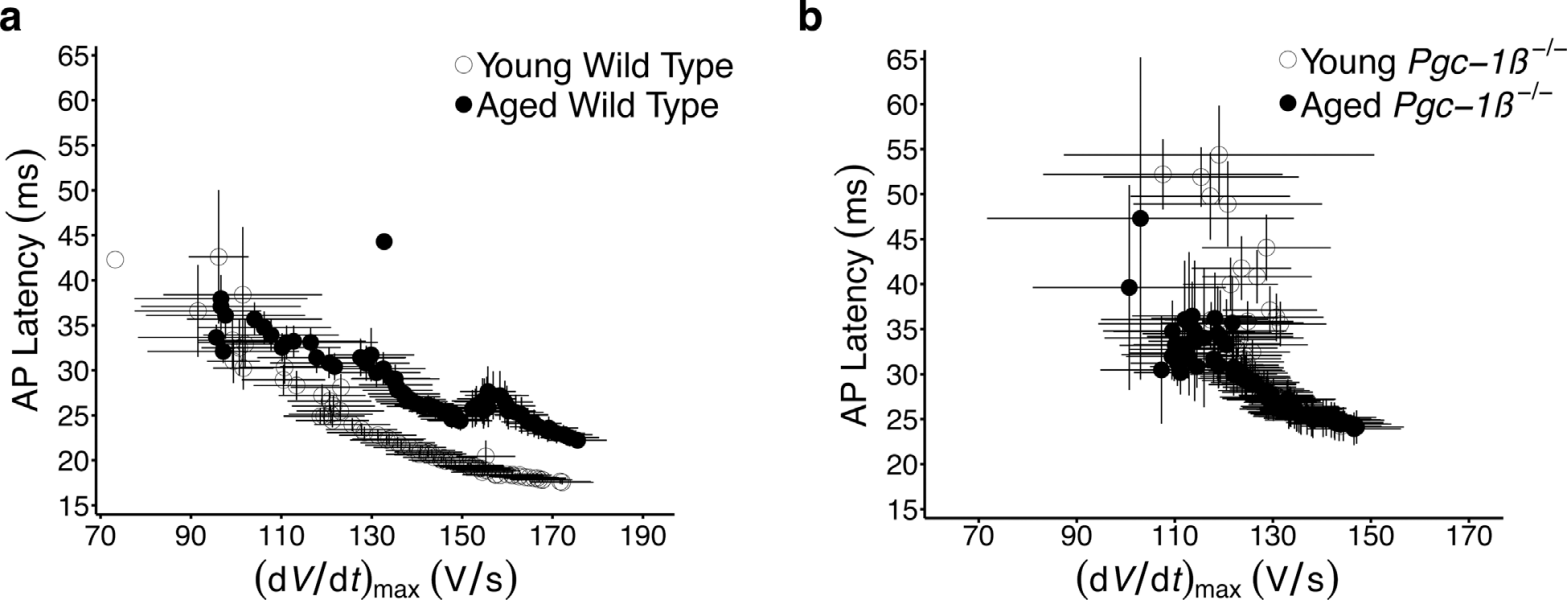
Dependences of AP latency times upon (d*V*/d*t*)_max_ through the programmed electrical stimulation protocol compared in (a) young and old WT and (b) young and aged *Pgc-1β^−/−^* hearts.

Findings from the regular 8 Hz and PES pacing protocols suggested an arrhythmic substrate in *Pgc-1β^−/−^* hearts through compromised conduction parameters, with few alterations in repolarisation characteristics. Here, *Pgc-1β^−/−^* hearts displayed deficits in (d*V*/d*t*)_max_ that were independent of age, and a corresponding altered conduction through the myocardium, reflected by prolonged AP latency times. However, the latter, differed from (d*V*/d*t*)_max_, in appearing to be influenced by age to some degree. This prompted further exploration of the relationship between these conduction parameters. Mean AP latency times from extrasystolic S2 beats recorded during the PES protocols are plotted against their corresponding mean (d*V*/d*t*)_max_ values for each experimental group in Fig. 7. Reductions in (d*V*/d*t*)_max_ with shortening S1-S2 coupling intervals is associated with increasing AP latency times for all groups, suggesting that much of the increase in AP latency observed with progressively shortening coupling intervals is attributable to concurrent reductions in (d*V*/d*t*)_max_. However differing associations between these parameters were seen in WT and *Pgc-1β^−/−^* hearts. As shown in Fig. 7(a), prolongation of AP latency independent of (d*V*/d*t*)_max_ occurs in WT hearts with age, such that for any given (d*V*/d*t*)_max_ value, the AP latency time is longer in aged WT hearts compared to young WT hearts. In contrast, young and aged *Pgc-1β^−/−^* hearts displayed a more homogeneous association between (d*V*/d*t*)_max_ and AP latency, with values in line with those of aged WT hearts. Thus young *Pgc-1β^−/−^* hearts develop electrophysiological features resembling those of normal ageing, which may explain their increased propensity to arrhythmia.

### 3.6 Compromised conduction triggering arrhythmia in all hearts

We next explored the bearing of the observed electrophysiological parameters upon the initiation of arrhythmic events. The mean values of the relevant electrophysiological parameters from the first S2 AP that triggered an episode of AT in a given heart are given in Table 5. *Pgc-1β^−/−^* hearts showed significantly higher values of (d*V*/d*t*)_max_ for triggering S2 APs compared to WT (*F* *=* 4.55, p < 0.05), but there were no effects of age (*F* *=* 0.00, p > 0.05) or interacting effects of age and genotype (*F* *=* 0.28, p > 0.05). No significant differences were found upon individual comparisons during post hoc analysis. Interestingly, ANOVA analysis of AP latencies for the same S2 trigger APs revealed no differences with respect to genotype (*F* *=* 0.001, p > 0.05), age (*F* *=* 0.15, p > 0.05) or interaction of the two (*F* *=* 3.46, p > 0.05). No significant differences were seen in APD_90_ between trigger S2 APs of the different experimental groups (ANOVA – genotype: *F* = 1.02, p > 0.05; age: *F* = 0.37, p > 0.05; interaction: *F* = 0.39, p > 0.05). Similarly AP wavelengths for trigger S2 APs were also indistinguishable between groups (ANOVA – genotype: *F* = 1.96, p > 0.05; age: *F* = 0.07, p > 0.05; interaction: *F* = 1.04, p > 0.05). Thus AT was initiated in WT and *Pgc-1β^−/−^* hearts through premature beats that were indistinguishable in terms of measures of conduction across the tissue. The differing profiles of conduction between the groups, and the earlier development of compromise in *Pgc-1β^−/−^* hearts may explain their increased vulnerability to arrhythmia.

**Table 5:** AP parameters for S2 triggered APs that initiated the first episode of atrial tachycardia during programmed electrical stimulation.

Experimental group	(dV/dt)_max_ (V s^−1^)	AP latency (ms)	APD_90_ (ms)	Wavelength
Wild Type Young	100.10 ± 5.68	27.42 ± 1.52	15.96 ± 0.50	1.58 ± 0.10
Wild Type Aged	112.53 ± 10.63	33.90 ± 3.39	17.60 ± 3.59	1.98 ± 0.44
Pgc-1β-/- Young	142.03 ± 11.77	32.86 ± 1.77	15.17 ± 0.61	2.12 ± 0.16
Pgc-1β-/- Aged	137.46 ± 14.65	28.57 ± 2.56	15.43 ± 0.85	2.04 ± 0.18

All values are given as mean (±SEM).

Symbols denote significant difference based on post hoc analysis, performed if the F value from two-way ANOVA was significant. Single, double and triple symbols denote p < 0.05, p < 0.01 and p < 0.001 respectively.

### 3.7 Increased fibrotic change with Pgc-1β ablation

The influence of ageing and genotype upon the relationship between rates of depolarisation and latency prompted histological assessment for fibrotic change. Fibrosis is known to impede AP conduction through the myocardium through decoupling of adjacent myocytes, resulting in disrupted gap junction functioning and consequent increases in resistivity. In addition, fibroblast fusion with myocytes increases membrane capacitance. Histological assessment was conducted blindly by two investigators independently and the ICC, a measure of consistency between their findings, was 0.88 suggesting a high degree of consistency in the results.

**Fig. 8:**
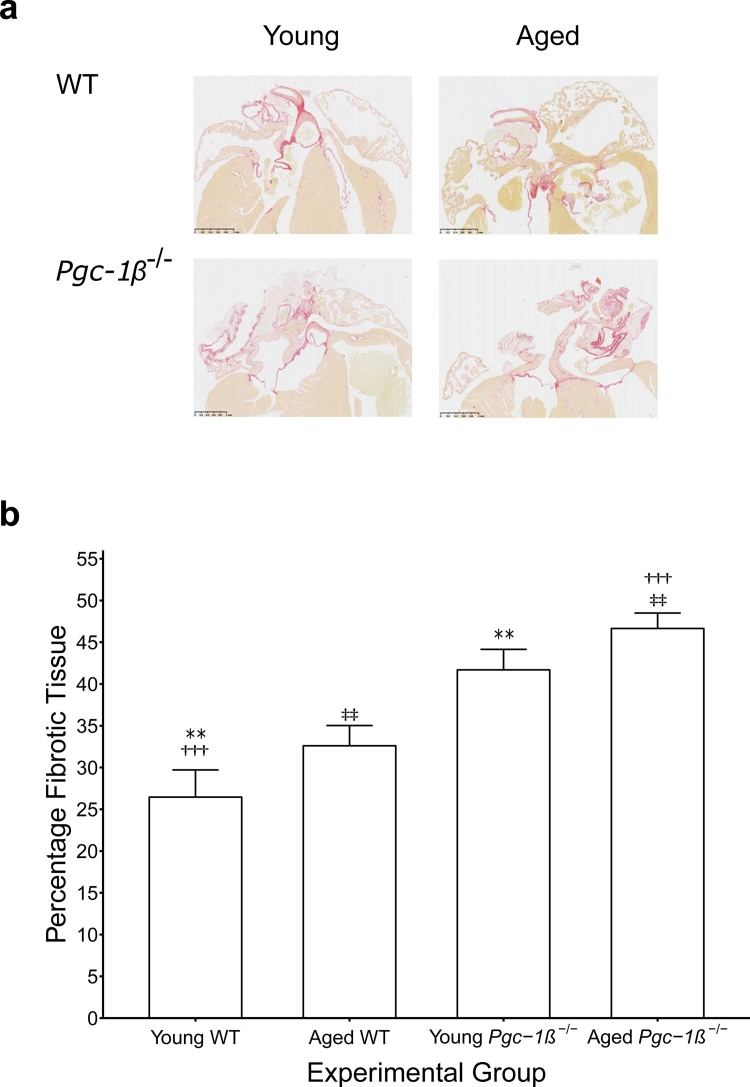
(a) Representative images of histological samples used for morphological assessment of fibrotic change. (b) The degree of fibrotic change was assessed as the proportion of morphometric squares covering tissue that demonstrated evidence of fibrosis as detected by picrosirius red staining. The numbers of hearts examined: young WT (n = 6), aged WT (n = 6), young *Pgc-1β^−/−^* (n = 6), aged *Pgc-1β^−/−^* (n = 6). Symbols denotes pairs of points showing significant differences from post hoc Tukey testing, where single, double and triple symbols denote p < 0.05, p < 0.01 and p < 0.001 respectively. (For interpretation of the references to colour in this figure legend, the reader is referred to the web version of this article).

Fig. 8(a) represents typical histological sections from young and aged WT and *Pgc-1β^−/−^* hearts, the quantification of fibrotic change is shown in Fig. 8(b). Genotype (*F* *=* 33.02, p < 0.001) and age (*F* *=* 4.75, p < 0.05) independently increases levels of fibrosis in the atria, but there was no evidence of interaction between the two. These findings complement the changes in latency noted in the earlier electrophysiological studies. The fibrotic change witnessed in WT aged hearts compared to WT young hearts explains the increased latency seen in this group. Young *Pgc-1β^−/−^* hearts show similar levels of fibrosis to aged WT hearts further supporting the suggestion of premature ageing in this group.

## 4 Discussion

Atrial fibrillation is characterised by an irregular, often rapid atrial rhythm that may be asymptomatic in the short term, but nevertheless carries substantial risks of long term morbidity and mortality. Ageing represents the major risk factor for AF itself: AF affects <0.1% in those under the age of 50, 4% in individuals aged 60–69 and up to 20% in those aged above 85 (Go et al., 2001, Krijthe et al., 2013, Zoni-Berisso et al., 2014). A decline in mitochondrial function is correspondingly observed in ageing, and has been postulated to account for age-related decrements in organ function with associated susceptibility to disease (Biala et al., 2015, Lane et al., 2015), including predisposition to AF (Ad et al., 2005, Montaigne et al., 2013, Tsuboi et al., 2001). The present study therefore investigated the electrophysiological alterations associated with ageing and mitochondrial dysfunction in murine atria with homozygous deficiency of the transcriptional co-activator *Pgc-1β.* We report an age-related increase in arrhythmic incidence that was exacerbated by mitochondrial dysfunction. This propensity to arrhythmia developed predominantly through deficits in parameters pertaining to AP conduction at the cellular and tissue level.

The heart is a highly oxidative organ and served by a rich network of mitochondria, which account for up to 30% of myocardial volume and produce approximately 95% of its cellular ATP (Schaper et al., 1985). Understandably mitochondrial dysfunction is associated with altered cardiac electrical properties, giving rise to AP heterogeneities that provide a substrate for arrhythmia, and has been the subject of much attention in the context of ventricular arrhythmogenesis (Brown and O’Rourke, 2010, Yang et al., 2014). Mitochondrial abnormalities have also been reported in studies of AF but their role in its pathogenesis have been relatively unexplored. Evidence of altered mitochondrial structure was found in dog (Morillo et al., 1995) and goat (Ausma et al., 1997) models of AF. Here, AF was induced by rapid atrial pacing with the noted mitochondrial defects appearing as a possible consequence of the pacing protocol and/or AF itself. Similarly, evidence of mitochondrial abnormalities have been reported in tissue samples obtained during cardiac surgery from AF patients (Emelyanova et al., 2016, Lin et al., 2003, Slagsvold et al., 2014, Tsuboi et al., 2001). In these studies the selected cohorts had established AF and it remains difficult to distinguish whether the observed mitochondrial lesions were caused by or resulted from AF, or indeed were a confound of ageing or other age-related conditions associated with metabolic compromise. However baseline mitochondrial deficits were found to predict development of post-operative AF following cardiac surgery in patients with no prior history of AF (Ad et al., 2005, Montaigne et al., 2013), suggesting a more direct role in its pathogenesis. Furthermore, Marks and colleagues recently reported that diastolic Ca^2+^ leak, through progressive oxidation of ryanodine receptors, was associated with age-dependent development of AF in a murine model (Xie et al., 2015). Reductions in mitochondrial ROS production attenuated these diastolic Ca^2+^ transients and prevented AF.

Electrophysiological alterations secondary to chronic mitochondrial impairment have not been well characterised to date. The PGC-1 family of transcriptional coactivators, which includes PGC-1α and PGC-1β, serve as key modulators of cellular metabolic activity, particularly in oxidative tissues such as the heart and brain (Riehle and Abel, 2012). Their present study combines the hitherto separate use of murine cardiac models in metabolic and electrophysiological studies. First, these demonstrated that *Pgc-1α* and *Pgc-1β* murine models do replicate the clinically reported energetic changes. Both *Pgc-1α-/-* (Arany et al., 2005, Huss et al., 2004, Vega et al., 2000) and *Pgc-1β-/-* mice (Lelliott et al., 2006) showed blunted expression of oxidative phosphorylation genes and reduced mitochondrial enzymatic activities, capacities for mitochondrial ATP synthesis and cytosolic ATP levels. Conversely, overexpression in specific *Pgc-1* family members increased expression of nuclear and mitochondrial genes, mitochondrial density and their oxidative capacity (Lehman et al., 2000, Russell et al., 2004).

Secondly, murine models have previously successfully replicated and clarified human phenotypes in arrhythmogenic conditions involving monogenic ion channel disorders. Murine hearts have similar overall anatomies and capacities to generate polymorphic arrhythmia (Martin et al., 2011, Matthews et al., 2013, Matthews et al., 2012, Matthews et al., 2010, Vaidya et al., 1999) involving formation of drifting rotors generating scroll waves even in their small volumes of tissue, as in human hearts (Gray et al., 1995). Mouse cardiac, particular ventricular AP waveforms show shorter, triangulated recovery waveforms distinct from those in humans, reflecting differing contributions from the various K^+^ current subtypes. Nevertheless, APs of mouse atria and ventricles resemble those of human hearts in their rapid depolarisation phases driven by inward *I*_Na_ (Guo et al., 1999) and their resulting transmural AP conduction velocities (Higuchi and Nakaya, 1984, Liu et al., 2004). Monogenic murine cardiac models successfully demonstrated clinically observed atrial and ventricular arrhythmic phenotypes and their mechanisms following loss of *Scn5a* function replicating Brugada Syndrome and following challenge by class I, Na^+^ channel blocking drugs, both situations compromising AP conduction (Dautova et al., 2010, Guzadhur et al., 2013, Martin et al., 2012).

Thirdly, deficiencies in either or both *Pgc-1α* and *Pgc-1β* do induce cardiac phenotypes. Mice deficient in both genes develop a lethal low cardiac output state and conduction system disease (Lai et al., 2008). Finally, *Pgc-1β* deficiency is not associated with cardiac dysfunction at baseline (Lelliott et al., 2006), but is associated with increased susceptibility to ventricular arrhythmias (Gurung et al., 2011). This makes it an appropriate model to investigate electrophysiological alterations secondary to mitochondrial dysfunction in murine atria. The modified Langendorff preparation utilised here permitted simultaneous volume conductor ECG and intracellular microelectrode recordings during regular pacing and programmed electrical stimulation applying premature extra stimuli, enabling assessment of AP activation and recovery properties

Hearts were first paced at a frequency of 8 Hz, reflecting murine resting heart rates and therefore providing steady state electrophysiological characterisation. No arrhythmias were observed in any group during regular pacing. This is consistent with previous reports that *Pgc-1β* ablation is not associated with a pronounced cardiac phenotype under conditions of baseline activity. In contrast arrhythmias were seen in all experimental groups during programmed electrical stimulation. Incidences of arrhythmia increased with age in both WT and *Pgc-1β^−/−^* hearts, in keeping with the cumulative risk of both atrial and ventricular arrhythmias with age seen in the clinical setting (Deo and Albert, 2012, Zoni-Berisso et al., 2014). Young WT hearts displayed the fewest episodes of AT of all groups, and the incidence was higher in aged WT hearts. The mitochondrial theory of ageing posits progressive deterioration in mitochondrial function, through accumulation of mutations in mitochondrial DNA and impaired autophagy, underpinning the ageing process and may contribute to this increased vulnerability to arrhythmia. Accordingly *Pgc-1β^−/−^* hearts, possessing a pronounced mitochondrial defect, had even higher incidences of AT. Here aged *Pgc-1β^−/−^* hearts displayed the greatest propensity to arrhythmia of all groups, in terms of the proportion of hearts that were arrhythmic and the overall number of episodes of arrhythmia.

The electrophysiological alterations underlying the greater propensity to arrhythmia in *Pgc-1β^−/−^*^−^ hearts were examined with intracellular AP recordings from the left atrium, which suggested that these occurred primarily through abnormalities in AP conduction. At the cellular level, young and aged *Pgc-1β^−/−^* hearts had significantly reduced (d*V*/d*t*)_max_ values compared to WT hearts during regular pacing. There was no difference in (d*V*/d*t*)_max_ based upon age in either group. A similar pattern was also observed in APs triggered by S2 stimuli during the PES protocol. As would be expected, (d*V*/d*t*)_max_ values progressively reduced with shortening of the coupling interval in all groups. At the longest coupling intervals (d*V*/d*t*)_max_ values differed between WT and *Pgc-1β^−/−^* hearts to similar extents as during 8 Hz pacing, whereas they converged to become indistinguishable at the shortest coupling intervals. Thus *Pgc-1β^−/−^* hearts demonstrated compromise at modest levels of stress represented by the longer coupling intervals, and correlated with their increased susceptibility to arrhythmia through greater parts of the protocol.

Reduced atrial conduction velocities have been reported as an early feature in patients with AF (Zheng et al., 2016) and potentially play a significant role in providing a substrate for its maintenance in the long term (Miyamoto et al., 2009, Park et al., 2009). Values of (d*V*/d*t*)_max_ are known to correlate with peak Na^+^ currents (*I*_Na_) (Hondeghem and Katzung, 1977) and conduction velocity in skeletal and cardiac cells (Fraser et al., 2011, Usher-Smith et al., 2006). Thus, *I*_Na_ dominates over Ca^2+^ current, *I*_Ca_, as the dominant inward, depolarising, current in atrial cardiomyocytes. It accounts for significantly greater maximum conductances (7.8 nS/pF vs. 0.12 nS/pF (Van Wagoner et al., 1999)) and maximum currents (∼85 pA/pF (Burashnikov et al., 2007, Sossalla et al., 2010) vs. ∼6 to 9 pA/pF (Carnes et al., 2007, Courtemanche et al., 1998)), at voltages corresponding to the most rapidly rising phase of the action potential (–30 mV vs +10 mV (Van Wagoner et al., 1999)). Time-dependent reductions in *I*_Na_ and consequent reductions in atrial conduction velocity have previously been implicated in the pathogenesis in a canine model of AF (Gaspo et al., 1997a). Furthermore *SCN5A* gene variants, which encodes the cardiac sodium channel responsible for the inward Na^+^ current, are associated with increased risk of developing AF (Darbar et al., 2008, Olson et al., 2005).

Mitochondrial dysfunction can alter *I*_Na_ through a number of potential mechanisms. Firstly reductions in *I*_Na_ in cardiomyocytes were observed in response to metabolic stress (Liu et al., 2009) and could be recovered with application of the mitochondrial ROS scavenger mitoTEMPO (Liu et al., 2010). Secondly, fluctuations in cytosolic [Ca^2+^] could also potential modify sodium channel properties through binding in its C-terminal region, either directly at an EF hand motif (Wingo et al., 2004) and indirectly through an IQ domain sensitive to calmodulin/calmodulin kinase II (Mori et al., 2000). Elevated intracellular [Ca^2+^] caused reductions in *I*_Na_ density and (d*V*/d*t*)_max_ in cardiomyocytes in vitro (Casini et al., 2009), and in whole hearts following diastolic Ca^2+^ leaks, through application of caffeine (Zhang et al., 2009), known to increase diastolic Ca^2+^ release, or mutations associated with diastolic Ca^2+^ release (Glukhov et al., 2013, King et al., 2013b, Li et al., 2014). Abnormal diastolic Ca^2+^ transients have been recorded in cardiomyocytes in *Pgc-1β^−/−^* hearts (Gurung et al., 2011).

The conduction of an AP wavefront through tissue is influenced by the membrane capacitance and its resistance, in addition to (d*V*/d*t*)_max_ (Jeevaratnam et al., 2011, King et al., 2013a). Conduction was therefore further assessed through evaluation of AP latency times. These were significantly prolonged in *Pgc-1β^−/−^* hearts compared to WT during regular pacing, with young WT hearts having significantly shorter AP latency durations than any other experimental group including aged WT hearts. During PES pacing, AP latency times increased with shortening of the S1-S2 coupling interval in all groups, but with differing magnitudes. Conduction slowing was most pronounced in *Pgc-1β^−/−^* hearts, particularly aged *Pgc-1β^−/−^* hearts, at the shorter coupling intervals correlating with their greater vulnerability to arrhythmia during the PES protocols. The differing comparisons of (d*V*/d*t*)_max_ and AP latency between groups were further explored by evaluating the dependence of AP latency upon (d*V*/d*t*)_max_ within groups. In all cases AP latency lengthened with reductions in (d*V*/d*t*)_max_, in keeping with the known relationship between (d*V*/d*t*)_max_, *I*_Na_ and conduction velocity (Hunter et al., 1975). However young and aged WT hearts displayed distinct relationships between AP latency and (d*V*/d*t*)_max_, with age-related delays in latency observed at any given (d*V*/d*t*)_max_ value. In contrast this correlation was indistinguishable between young and aged *Pgc-1β^−/−^* hearts, where both resembled the conduction slowing seen with ageing in WT hearts.

Myocardial fibrosis is associated with increased tissue capacitance and resistance, contributing to conduction slowing independent of the influence of (d*V*/d*t*)_max_ and may explain the conduction properties described in the present study. Fibrotic change is thought to be a key element of the remodeling seen in AF (Frustaci et al., 1997, Kostin et al., 2002). Progressive fibrosis is a common feature of cardiac ageing in animal (Eghbali et al., 1989, Jeevaratnam et al., 2012, Lin et al., 2008, Orlandi et al., 2004) and human (Gazoti Debessa et al., 2001) studies. Age-dependent fibrosis was similarly recorded in the present study, in both WT and *Pgc-1β^−/−^* hearts, and mitochondrial dysfunction through *Pgc-1β* ablation was associated with a further additive effect on the degree of fibrosis. Aged *Pgc-1β^−/−^* hearts therefore displayed the greatest degree of fibrosis whereas young WT hearts had the least and AP latency times correlated with the observed degrees of fibrosis in the respective groups.

The fibrosis-mediated reductions in cardiac conduction reported here could potentially occur through increased coupling of fibroblasts to cardiomyocytes through Cx43 and/or Cx45, thereby increasing membrane capacitance (Camelliti et al., 2004, Chilton et al., 2007, Van Veen et al., 2005). More direct disruption of gap junctions has also been reported consequently increasing tissue resistance, further slowing conduction (Xie et al., 2009). Interestingly, mice lacking the mitochondrial sirtuin SIRT3 display augmented mitochondrial ROS production and enhanced cardiac fibrosis (Hafner et al., 2010). Upregulated antioxidant capacity through a mitochondrial specific overexpression of catalase protected against features of cardiac ageing including myocardial fibrosis (Dai et al., 2009). Furthermore, transforming growth factor-β1 (TGF-β1) is thought to have a significant role in age-related myocardial fibrosis (Brooks and Conrad, 2000, Rosenkranz et al., 2002). Mice over expressing TGF-β_1_ develop pronounced atrial fibrosis, have reduced atrial conduction velocities and greater inducibility to atrial tachyarrhythmias including AF (Verheule et al., 2004). Serum levels of TGF-β_1_ are increased in individuals with AF compared to control (Lin et al., 2015). TGF-β_1_ activity is also enhanced by oxidative stress (Barcellos-Hoff and Dix, 1996, Sullivan et al., 2008).

A pro-arrhythmic substrate can also develop through altered repolarisation properties including reductions in the APD or shortening of the atrial ERP. Reductions in APD have been documented in AF and were also seen in the present study, consistent with the previously reported effect of mitochondrial dysfunction upon AP repolarisation properties (Brown and O’Rourke, 2010). However these were witnessed in aged *Pgc-1β^−/−^* hearts and most pronounced at shorter S1–S2 coupling intervals in the PES protocol and would favour re-entry and arrhtymogenesis. These parameters pertaining to AP recovery can be combined with those of AP activation to give the AP wavelength, defined as the distance travelled by the depolarising wave over one refractory period (Allessie et al., 1977). Shortening of the AP wavelength favours re-entry whereas its lengthening is thought to be protective (Davidenko et al., 1995, Pandit and Jalife, 2013, Spector, 2013, Weiss et al., 2005, Zaitsev et al., 2000) AP wavelength was shorter in *Pgc-1β^−/−^* hearts than WT heart, both at circumstances mimicking resting heart rates and PES pacing. With reductions in the S1-S2 coupling interval, the shorter AP wavelengths in aged *Pgc-1β^−/−^*hearts persisted, whereas those for young *Pgc-1β^−/−^* hearts and WT hearts overlapped, correlating with the differing arrhythmic susceptibilities observed in the present study.

Finally, the influence of these electrophysiological parameters upon arrhythmia induction was examined in the first S2 AP that provoked an episode of AT in each heart. Interestingly (d*V*/d*t*)_max_ values were higher for S2 beats triggering AT in *Pgc-1β^−/−^* hearts than WT hearts; however no difference was seen in AP latency times. Thus the higher (d*V*/d*t*)_max_ values of *Pgc-1β^−/−^* hearts appear to offset the increased fibrotic changes in these hearts, which therefore achieve a similar overall conduction velocity. Similarly, no difference in APD_90_ or AP wavelength were seen in S2 beats triggering arrhythmias in WT and *Pgc-1β^−/−^* hearts. This suggested critical electrophysiological thresholds common to all groups, below which the susceptibility to arrhythmias is increased. The differing parameters measured in the current experiments indicate these circumstances arise earlier through ageing and mitochondrial dysfunction, thus widening the range of S1-S2 intervals at which the *Pgc-1β^−/−^* hearts were at risk of arrhythmia compared to WT hearts. Accordingly, the critical coupling intervals were longer in *Pgc-1β^−/−^* hearts compared to WT hearts, and in particular in aged *Pgc-1β^−/−^* hearts.

## 5 Conclusion

Together, the present experiments associate an atrial arrhythmic phenotype secondary to a chronic mitochondrial deficit, modeled by in murine *Pgc-1β^−/−^* hearts. This is attributable to maladaptive alterations in action potential conduction arising from electrophysiological changes at the cellular level and structural, fibrotic, changes at the tissue level.

## Conflicts of interest

None declared.
